# A Novel Monoclonal Anti-CD81 Antibody Produced by Genetic Immunization Efficiently Inhibits Hepatitis C Virus Cell-Cell Transmission

**DOI:** 10.1371/journal.pone.0064221

**Published:** 2013-05-21

**Authors:** Isabel Fofana, Fei Xiao, Christine Thumann, Marine Turek, Laetitia Zona, Rajiv G. Tawar, Fritz Grunert, John Thompson, Mirjam B. Zeisel, Thomas F. Baumert

**Affiliations:** 1 Inserm, U1110, Institute of Virology, Strasbourg, France; 2 University of Strasbourg, Strasbourg, France; 3 Aldevron GmbH, Freiburg, Germany; 4 Pôle Hépato-digestif, Hôpitaux Universitaires de Strasbourg, Strasbourg, France; Inserm, France

## Abstract

**Background and Aims:**

Hepatitis C virus (HCV) infection is a challenge to prevent and treat because of the rapid development of drug resistance and escape. Viral entry is required for initiation, spread, and maintenance of infection, making it an attractive target for antiviral strategies.

**Methods:**

Using genetic immunization, we produced four monoclonal antibodies (mAbs) against the HCV host entry factor CD81. The effects of antibodies on inhibition of HCV infection and dissemination were analyzed in HCV permissive human liver cell lines.

**Results:**

The anti-CD81 mAbs efficiently inhibited infection by HCV of different genotypes as well as a HCV escape variant selected during liver transplantation and re-infecting the liver graft. Kinetic studies indicated that anti-CD81 mAbs target a post-binding step during HCV entry. In addition to inhibiting cell-free HCV infection, one antibody was also able to block neutralizing antibody-resistant HCV cell-cell transmission and viral dissemination without displaying any detectable toxicity.

**Conclusion:**

A novel anti-CD81 mAb generated by genetic immunization efficiently blocks HCV spread and dissemination. This antibody will be useful to further unravel the role of virus-host interactions during HCV entry and cell-cell transmission. Furthermore, this antibody may be of interest for the development of antivirals for prevention and treatment of HCV infection.

## Introduction

Hepatitis C virus (HCV) is a major cause of chronic hepatitis worldwide. The current therapy against HCV infection based on pegylated interferon-alfa (PEG-IFN-α) and ribavirin does not allow to cure all patients. Although the addition of a direct-acting antiviral (DAA) targeting HCV protein processing - telaprevir or boceprevir- to the standard of care improves sustained virological response in genotype 1 infected patients, toxicity of the individual compounds and development of viral resistance remain major challenges [1]. To date, a vaccine is not available and the absence of preventive strategies is a major limitation for patients undergoing liver transplantation (LT) for HCV-related end-stage liver disease. Re-infection of the graft is universal and characterized by accelerated progression of liver disease [2]. Efficacy and tolerability of IFN-based therapies are limited in LT recipients [3], [4] and potentially life-threatening drug-drug interactions limit the use of DAAs in these patients if combined with immunosuppressive agents [5]. Thus, there is an urgent need for novel antiviral preventive and therapeutic strategies.

HCV entry is a multifactorial process involving several host cell factors, including the four main entry factors CD81, scavenger receptor class B type I (SR-BI), claudin-1 (CLDN1) and occludin (OCLN), as well as co-entry factors such as epidermal growth factor receptor (EGFR), ephrin receptor A2 (EphA2), and the Niemann-Pick C1-Like 1 (NPC1L1) cholesterol absorption receptor [6], [7]. This process thus provides numerous targets for antivirals. Targeting viral entry offers the advantage to combat viral infection at the very first steps of virus infection and before the virus starts to produce genomic material that will persist in infected cells. Proof-of-concept studies showed that entry inhibitors efficiently prevent or delay HCV infection *in vitro* and *in vivo* [6]. Viral entry inhibitors are thus unique and feasible antiviral candidates to prevent HCV infection in transplant recipients where entry has been shown to be a key determinant for infection of the liver graft [8], [9]. Furthermore, since entry is also required for dissemination and maintenance of infection [10], this approach may allow treating persistent infection as well.

CD81 is a member of the tetraspanin family of proteins, containing a small extracellular and a large extracellular loop (LEL). CD81 was the first HCV host factor to be identified by its ability to interact with a soluble form of HCV E2 (sE2) [11]. The HCV-CD81 interaction and its role in HCV infection have then been extensively studied using various model systems. The CD81 LEL plays an important role in this process [12], [13]. CD81 is an essential HCV host factor as silencing of CD81 expression in hepatoma cells inhibits HCV entry while CD81 expression in HCV-resistant hepatoma cell lines confers susceptibility to HCV entry [14], [15], [16], [17]. Although CD81 binds sE2 *in vitro*, it has a central role in HCV entry of viral particles during post-binding steps [18], [19], [20]. Indeed, CD81 associates with CLDN1 to form co-receptor complexes that are crucial for HCV internalization [20], [21], [22] and disruption of these complexes prevents HCV infection [23], [24], [25]. CD81 contributes to the species specificity of HCV infection as mouse cell lines and mouse hepatocytes become permissive to HCV entry upon expression of human CD81 and OCLN *in vitro* and *in vivo* [26], [27]. Furthermore, HCV mutants able to use mouse CD81 for cell entry have also been described [28]. Noteworthy, studies demonstrating that anti-CD81 antibodies can prevent HCV infection using uPA-SCID mice underscore the relevance of targeting CD81 for prevention of HCV infection [29].

In addition to cell-free virus entry, where CD81 has been described as an essential factor [12], [14], [15], [16], [17], [30], [31], HCV uses direct cell-cell transfer to infect neighbouring cells and persist in the presence of virus-neutralizing antibodies [10]. This process also seems to require several HCV host factors including CD81, SR-BI, CLDN1, OCLN, EGFR, EphA2 and NPC1L1 [10], [25], [32], [33] but has been less extensively characterized than cell-free entry. Although a CD81-independent route of HCV spread has been described [10], [34], [35], the exact role of CD81 in viral cell-cell transmission remains unknown.

In this study, we produced and functionally characterized a novel panel of monoclonal antibodies (mAbs) directed against CD81 generated by genetic immunization which specifically and dose-dependently inhibit HCV infection at post-binding steps of the viral entry process. In addition to inhibiting cell-free HCV infection, one antibody was also able to completely block neutralizing antibody-resistant HCV cell-cell transmission and viral dissemination.

## Materials and Methods

### Cell lines

Culture of Huh7.5.1 [31], Huh7.5-GFP+ [34], HEK 293T [36], Chinese hamster ovary (CHO) [24] and HepG2 [37] cells has been described.

### Antibodies

Anti-CD81 mAbs were raised by genetic immunization of Wistar rats using an eukaryotic expression vector encoding the full-length human CD81 cDNA as previously described [9], according to proprietary Aldevron technology (Aldevron, Freiburg, Germany). Animal maintenance and immunization of rats to generate mAbs against CD81 were carried out by a certified animal facility in Germany (MfD Diagnostics GmbH) according to DIN EN ISOO 9000∶2000 standards, the regulations of the German Animal Act of 18 May 2006 (BGBI. I S. 1206) and the regulations of European Union guidelines 86/609/EWG of 24 November 2006 and according to the European Agreement of 18 March 1986 for protection of animal trials and other for scientific purposes used vertebrates (Act of 11 December 1990 (BGBI. II S. 1486). The protocol was reviewed by the MfD Diagnostics GmbH animal care committee. For immunization, the animals were anaesthetized using isofluorane. This standard technology does not create animal discomfort. The animals were sacrificed by trained personnel by CO_2_ gas and their draining lymph nodes removed as sources for the antibody-producing B-lymphocytes. Immediately following animal death, final bleeds were carried out by cardiopuncture. Antibodies were selected by flow cytometry for their ability to bind to human CD81 expressed on the cell surface of CHO cells transfected with pcDNA3.1-hCD81. Mouse anti-CD81 JS81 antibody was obtained from BD Biosciences. Rat anti-SR-BI (QQ-6G9-A6) and control rat mAbs have been reported [38]. Anti-E2 (IGH461, Innogenetics; AP33, Genentech; CBH23, a kind gift from S.K.H. Foung) mAbs and human anti-HCV IgG have been described [36], [39]. NS5A-specific antibody (Virostat), anti-rat IgG alkaline phosphatase (AP) antibody, phycoerythrin (PE)-anti-rat antibody have been described [25], [34].

### Binding to cell surface CD81

CHO cells were transduced with lentiviruses to express hCD81 and selected with 250 µg/ml of G418 [40]. HepG2 cells were transfected with a plasmid to express hCD81 and selected with 80 µg/ml of hygromycin [24]. Cells were then analyzed by flow cytometry for CD81 expression. Briefly, 2×10^5^ cells were stained with mAbs specific for hCD81 (monoclonal rat TN-4H4-F11, TN-9H6-D10, TN-5C5-F3, QV-6A8-F2-C4, 20 µg/ml) or with control mAb in PBS for 1 h at room temperature. Primary bound antibodies were detected with secondary polyclonal antibodies coupled to phycoerythrin (Beckman coulter, 1/100) for 45 minutes at 4°C in PBS. After washing, the cells were fixed with 2% PFA and analyzed by flow cytometry (BD LSR II Flow Cytometer) [38]. Results are expressed as net mean fluorescence intensities (ΔMFI).

### HCVcc and HCVpp production and infection

HCVcc (Luc-Jc1 and Jc1) [18] and HCVpp (H77, HCV-J, JFH1, UKN3A1.28, UKN4.21.16, UKN5.14.4, UKN6.5.340, P02VJ) [8], [41] were produced as described [8], [25]. Patient-derived HCVpp were produced from serum of a patient undergoing LT using full-length E1E2 expression constructs generated from circulating HCV as described [8], [36]. The study was approved by the Strasbourg University Hospital Institutional Review Board and written informed consent was obtained from all patients (ClinicalTrial.gov Identifier: NCT00213707). Huh7.5.1 cells were pre-incubated with antibodies for 1 h and then incubated with HCVpp or HCVcc for 4 h at 37°C. Analysis of viral infection was performed by detection of luciferase activity as described [18], [24], [25]. For combination experiments, anti-CD81 (QV-6A8-F2-C4) mAb was tested individually or in combination with the second antibody. Huh7.5.1 cells were pre-incubated with anti-CD81 or control mabs for 1 h and then incubated for 4 h at 37°C with HCVcc or HCVpp (pre-incubated for 1 h with or without anti-envelope antibodies) as described [38]. Synergy was assessed using the combination index (CI) [38], [42], [43]. A CI less than 0.9, between 0.9 and 1.1, and more than 1.1 indicates synergy, additivity, and antagonism, respectively [38], [42], [43]. Cell viability was assessed using a MTT test [9], [25].

### Kinetic assays

HCVcc kinetic entry assays were performed in Huh7.5.1 cells using anti-CD81 QV-6A8-F2-C4, anti-CD81 JS81, anti-SRBI QQ-4G9-A6 or control mAbs added at different time-points during or after viral binding as described [24], [38], [39].

### Cross-competition

Competition between anti-CD81 mAbs JS81 and QV-6A8-F2-C4 for cellular binding was measured by a cell-based ELISA and labeled antibodies: Huh7.5.1 or CHO-CD81 cells were incubated for 60 min with 0.1 µg/ml biotinylated JS81 (Sulfo-NHS-LC-Biotin; Thermo Scientific) together with increasing concentrations of unlabeled QV-6A8-F2-C4 as competitor. Following washing with PBS, binding of biotinylated antibody was detected by incubation with streptavidin labeled with horseradish peroxidase. Curves determined by measurement of binding in the presence of an isotype-matched control were compared to those determined in the presence of the competing antibody.

### Cell-cell transmission of HCV

Cell-cell transmission of HCV was assessed as described [25], [34]. Briefly, producer Huh7.5.1 cells were electroporated with HCV Jc1 RNA (Pi) and cultured with naive target Huh7.5-GFP+ cells (ratio of 1∶2) in the presence or absence of anti-CD81 or control mAbs (10 µg/ml). An HCV E2–neutralizing mAb (AP33, 25 µg/ml) was added to block cell-free transmission [34]. After 24 h of co-culture, cells were fixed with paraformaldehyde, stained with an NS5A-specific mAb (0.1 µg/ml) and analyzed by flow cytometry [25], [34]. Total and cell-cell transmission was defined as percentage HCV infection of Huh7.5-GFP+ target cells (Ti) in the absence (total transmission) or presence (cell-cell transmission) of an HCV E2-specific mAb [25].

### Immunofluorescence of viral dissemination

Virus spread was assessed by visualizing Jc1-infected Huh7.5.1 cells by immunofluorescence using anti-NS5A (Virostat) or anti-E2 (CBH23) mAbs as described [25]. In these long-term experiments, cells are plated and infected at low density and cell growth between control- and anti-CD81 antibody-treated cells was ascertained by enumeration of cells (by cell counting and by immunofluorescence staining of cell nuclei using DAPI) as described [38].

## Results

### Production of anti-CD81 monoclonal antibodies directed against cell surface CD81

**Figure 1:**
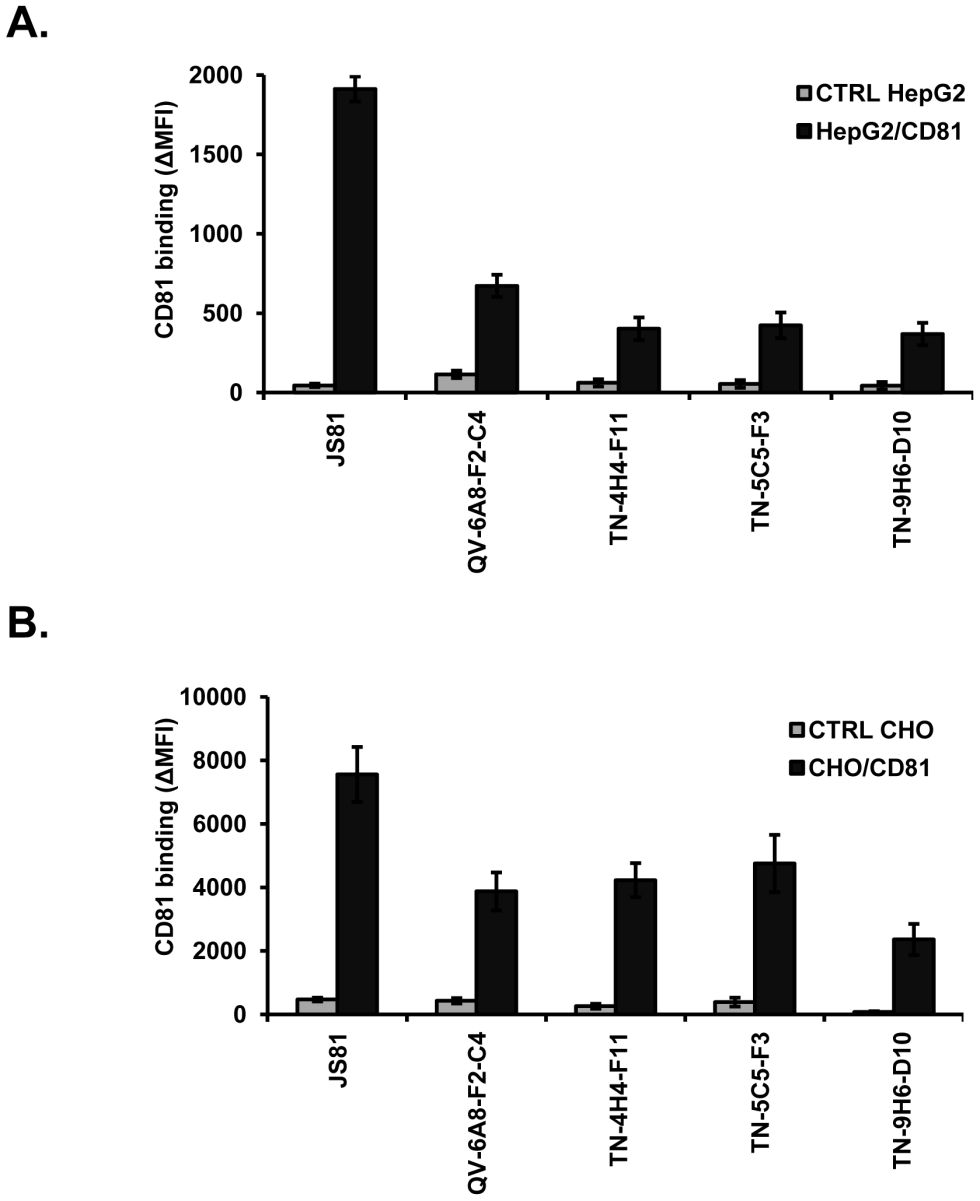
Production of CD81-specific mAbs directed against cell surface CD81. (A) HepG2 and HepG2 cells expressing human CD81 as well as (B) CHO and CHO cells expressing human CD81 were incubated with indicated anti-CD81 mAbs (20 µg/ml) and antibody binding was assessed using flow cytometry. Results are expressed as mean fluorescence intensity (ΔMFI) ± SEM of a pool of three independent experiments performed in duplicate.

To further explore CD81 as a target for antiviral strategies, we generated anti-CD81 mAbs by genetic immunization using a full-length human CD81 cDNA expression vector. Four mAbs (QV-6A8-F2-C4, TN-9H6-D10, TN-5C5-F3, TN-4H4-F11) were selected that reacted with native human CD81 expressed on HepG2 and CHO cells (Fig. 1A–B). To characterize the nature of the epitopes recognized by the four different anti-CD81 mAbs, we performed immunoblot analyses using Huh7.5.1 cells which express high levels of endogenous CD81 on their cell surface. Immunoblot analyses under reducing conditions demonstrated no staining of CD81 by anti-CD81 mAbs suggesting that the anti-CD81 mAbs most likely recognize predominantly conformational epitopes or their affinity to linear epitopes is low (data not shown).

### Inhibition of HCV infection by anti-CD81 monoclonal antibodies

**Figure 2:**
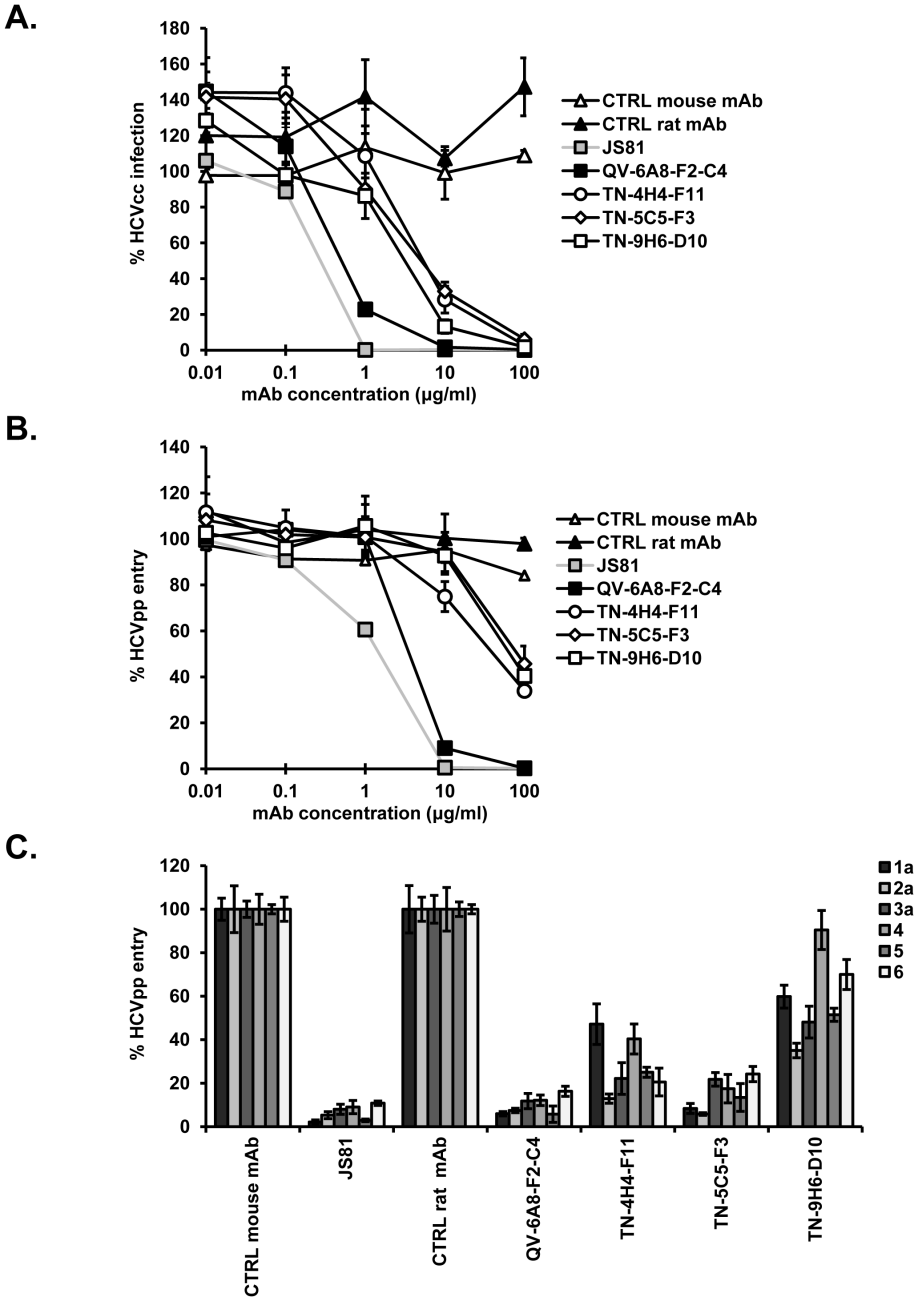
Anti-CD81 mAbs dose-dependently inhibit HCV infection. (A–B) Dose-dependent inhibition of HCV infection by anti-CD81 mAbs. Huh7.5.1 cells were pre-incubated with increasing concentrations of anti-CD81 or isotype control (CTRL IgG) mAbs for 1 h at 37°C before infection with (A) HCVcc (Luc-Jc1 (2a)) or (B) HCVpp (HCV-J (1b)). Three days later, viral infection was quantitated by assessing the expression of luciferase reporter gene. Results are expressed as % HCVcc infection or % HCVpp entry and represent means ± SD of one representative experiment performed in triplicate. (C) Inhibition of infection of HCVpp bearing envelope glycoproteins from genotypes 1–6. Huh7.5.1 cells were pre-incubated with a fixed concentration (100 µg/ml) of antibodies before infection with HCVpp (strains H77 (1a), JFH1 (2a), UKN3A1.28 (3a), UKN4.21.16 (4), UKN5.14.4 (5), UKN6.5.340 (6)). Means ± SD from a representative experiment performed in triplicate are shown.

To investigate whether these antibodies inhibit HCV infection, Huh7.5.1 cells were pre-incubated with anti-CD81 mAbs and infected with chimeric luciferase reporter virus Luc-Jc1 (genotype 2a). As shown in Fig. 2A, anti-CD81 mAbs inhibit Luc-Jc1 infection of Huh7.5.1 cells in a dose-dependent manner (IC_50_ of 0.7–8 µg/ml). In contrast, an isotype control mAb had no effect. Among these antibodies, anti-CD81 mAb QV-6A8-F2-C4 most efficiently inhibited HCVcc infection with an IC_50_ of 0.7 µg/ml. To investigate whether anti-CD81 mAbs were effective against other HCV genotypes, we analyzed their inhibition of HCVpp bearing envelope glycoproteins from HCV genotype 1b. All four anti-CD81 mAbs inhibited HCVpp genotype 1b entry in a dose-dependent manner (Fig. 2B). Anti-CD81 mAb QV-6A8-F2-C4, displaying the lowest IC_50_ against HCVcc from genotype 2, was also characterized by the lowest IC_50_ against HCVpp from genotype 1b (IC_50_ of 4 µg/ml). Inhibition of HCVcc infection and HCVpp entry by QV-6A8-F2-C4 was in a similar range as inhibition of infection by the commercially available anti-CD81 mAb JS81 (IC_50_s of 0.5 and 2 µg/ml, respectively). Interestingly, the IC_50_ of all these anti-CD81 mAbs were higher on inhibition of HCVpp entry than HCVcc infection, suggesting that these mAbs may act on another step of the viral life cycle in addition to cell-free entry. Noteworthy, these antibodies also blocked the infectivity of HCVpp bearing the envelope glycoproteins from HCV genotypes 2–6 (Fig. 2C). Taken together, these data indicate that anti-CD81 mAbs efficiently block HCV infection in a pan-genotypic manner.

### Anti-CD81 monoclonal antibody QV-6A8-F2-C4 inhibiting HCV infection targets post-binding steps of viral entry

**Figure 3:**
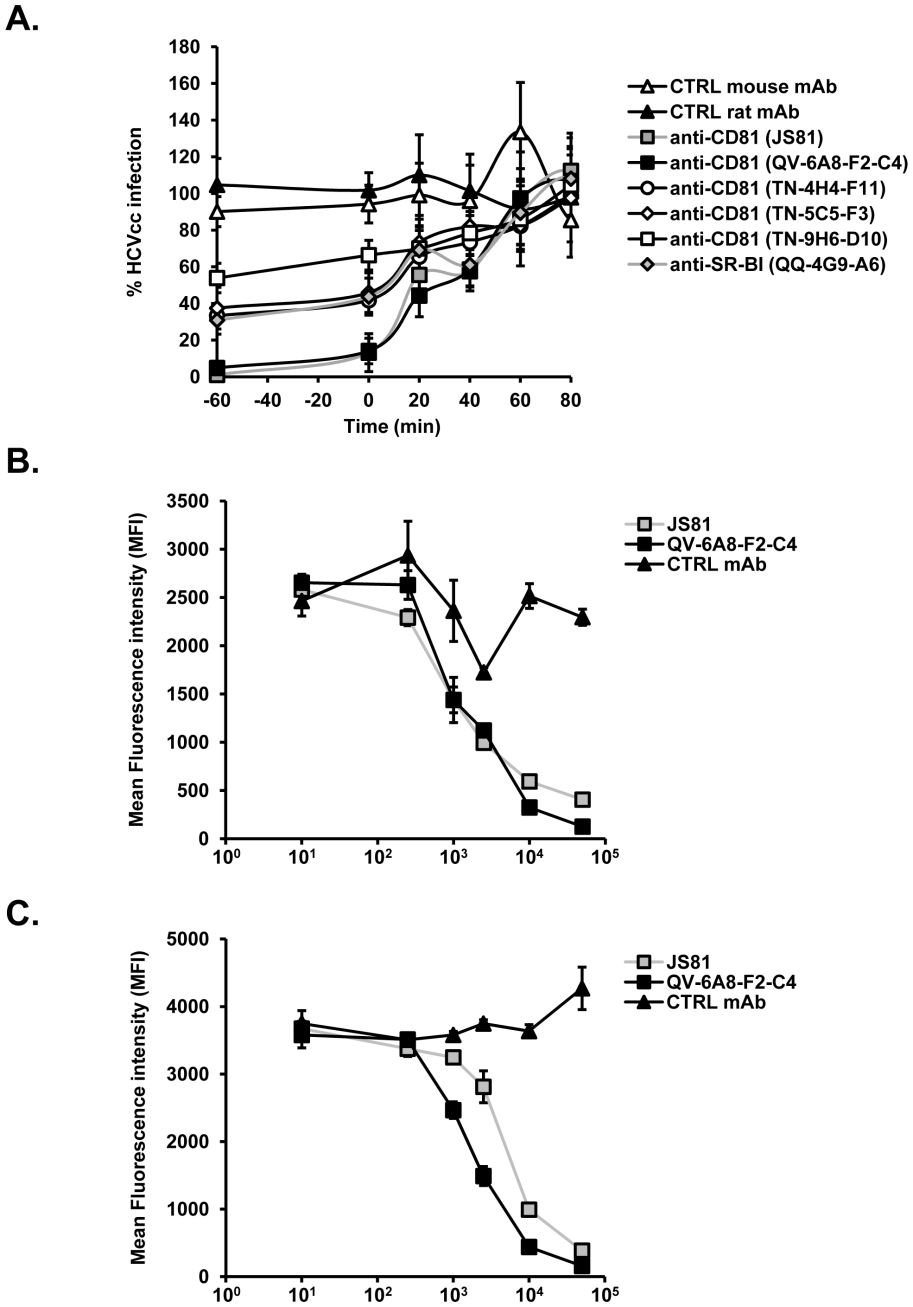
Anti-CD81 mAbs inhibit HCV infection at post-binding steps of viral entry. (A) Kinetics of HCVcc entry into human hepatoma cells. To discriminate between virus binding and post-binding events, Luc-Jc1 HCVcc binding to Huh7.5.1 cells was performed in the presence or absence of anti-CD81 mAbs QV-6A8-F2-C4, TN-9H6-D10, TN-5C5-F3 and TN-4H4-F11 (20 µg/ml), anti-CD81 mAb JS81 (5 µg/ml), anti-SR-BI mAb QQ-4G9-A6 (20 µg/ml) or respective control mAbs (20 µg/ml) for 1 h at 4°C, before cells were washed and incubated for 4 h at 37°C with mAbs added at different time-points during infection as described [24], [39]. Compounds were then removed and cells were cultured for an additional 48 h in the absence of mAbs before measuring HCV infection by luciferase assay. Results are expressed as % HCVcc infectivity relative to cells incubated in the absence of mAb and represent means ± SD from two independent experiments performed in triplicate. (B–C) Competition of anti-CD81 mAbs JS81 and QV-6A8-F2-C4 for cellular binding. (B) Huh7.5.1 or (C) CHO-CD81 cells were incubated with 0.1 µg/ml biotinylated anti-CD81 mAb JS81 together with increasing concentrations of unlabeled anti-CD81 mAb QV-6A8-F2-C4 as competitor. Following washing of cells in PBS, binding of labeled antibody was detected as described in Methods and is shown as mean fluorescence intensity (MFI).

CD81 has been demonstrated to participate in post-binding steps of the viral entry process [18], [19], [44]. To investigate the HCV entry steps targeted by our anti-CD81 mAbs, we investigated the inhibitory capacity of anti-CD81 mAbs in kinetic entry studies [24], [39], [45]. To allow virus binding, Luc-Jc1 HCVcc were first incubated with Huh7.5.1 cells for 1 h at 4°C in the presence or absence of antibodies. Then the temperature was shifted to 37°C to allow continuation of the viral entry process. Antibodies were added at different time-points after the temperature shift to assess their ability to inhibit the course of HCV entry. Anti-CD81 mAb JS81 and anti-SR-BI mAb QQ-4G9-A6, two antibodies that have been previously reported to inhibit HCV post-binding steps [18], [38], were used side-by-side. As shown in Fig. 3A, anti-CD81 mAb QV-6A8-F2-C4 inhibited HCVcc infection at post-binding steps similarly to results obtained with anti-CD81 mAb JS81 and anti-SR-BI mAb QQ-4G9-A6, while a control mAb had no effect. Noteworthy, cross-competition experiments on Huh7.5.1 and CHO-CD81 cells demonstrated that QV-6A8-F2-C4 and JS81 recognize similar epitopes on CD81 (Fig. 3B–C). Furthermore, anti-CD81 mAbs TN-9H6-D10, TN-5C5-F3 and TN-4H4-F11 also inhibited HCV entry at post-binding steps, albeit at lower levels (Fig. 3A). Taken together, these data indicate that anti-CD81 mAb QV-6A8-F2-C4 blocks HCV entry during post-binding steps.

### Synergy between anti-CD81 and anti-HCV envelope antibodies on inhibiting HCV escape variant infection

**Figure 4:**
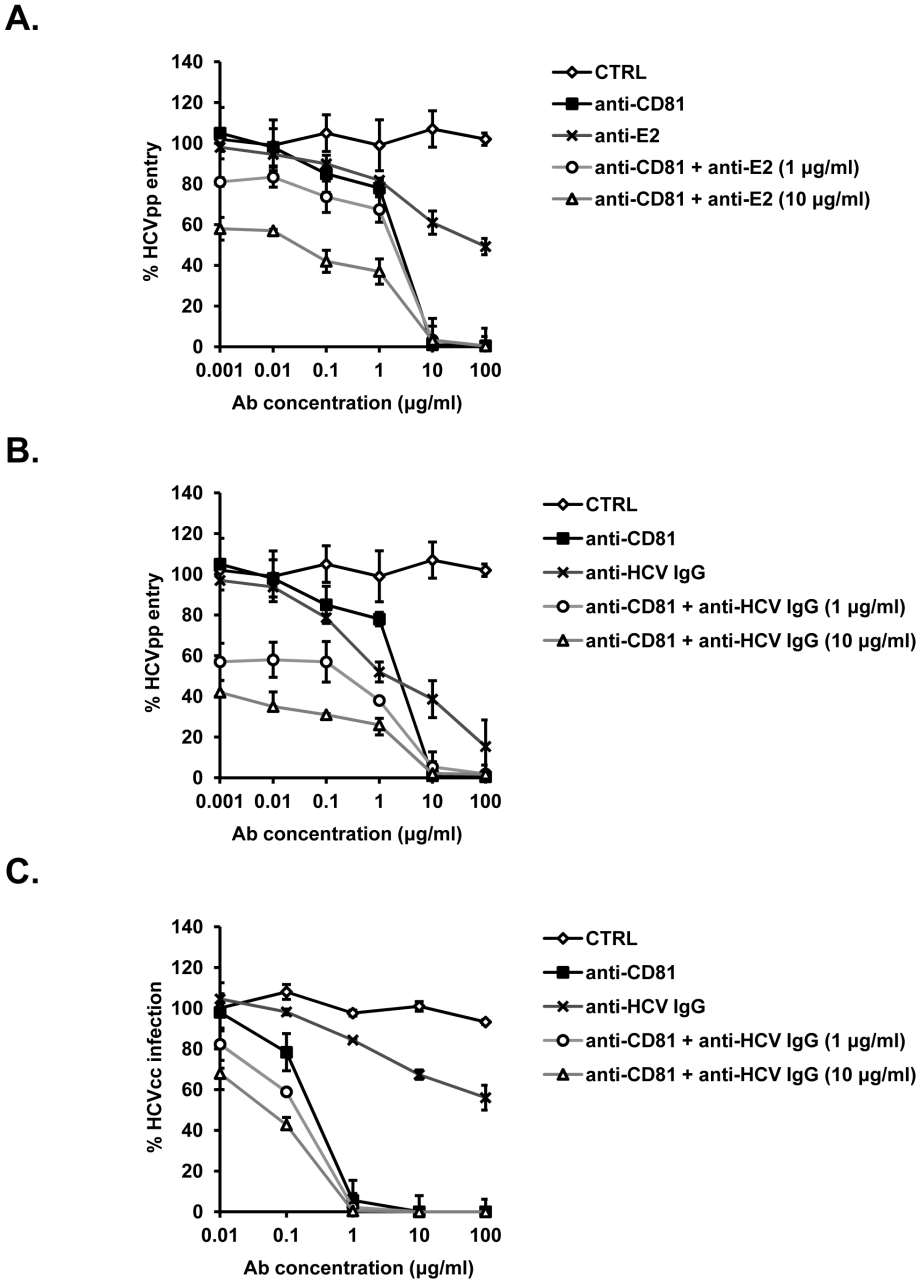
Synergy between anti-CD81 and anti-envelope antibodies in inhibiting HCV infection. (A–B) Patient derived HCVpp P02VJ or (C) HCVcc-Luc Jc1 were pre-incubated with (A) anti-E2 mAb (IGH461) or (B–C) purified heterologous anti-HCV IgG (1 or 10 µg/ml) obtained from an unrelated chronically infected subject or isotype control IgG for 1 h at 37°C and added to Huh7.5.1 cells that had been pre-incubated with increasing concentrations of control or anti-CD81 mAb QV-6A8-F2-C4. In anti-envelope antibody monotherapy setting, HCVpp or HCVcc were in parallel pre-incubated with increasing concentrations of anti-E2 mAb or anti-HCV IgG. HCVpp entry and HCVcc infection were analyzed by luciferase assay. Results are expressed as % HCVpp entry or HCVcc infection and represent means ± SD from a representative experiment performed in triplicate.

We have previously demonstrated that viral entry is a key determinant for HCV re-infection during LT and that HCV-CD81 interactions play an important role in this process [8], [46]. Moreover, we have demonstrated that receptor-specific antibodies or kinase inhibitors specifically inhibit entry of highly infectious HCV escape variants that are resistant to autologous host responses and re-infect the liver graft [8], [9], [25], [38]. To assess the clinical relevance of anti-CD81 mAbs to inhibit HCV escape variants, we determined the effect of anti-CD81 mAb QV-6A8-F2-C4 on entry into Huh7.5.1 cells of HCVpp bearing the envelope glycoproteins of a highly infectious HCV strain selected during LT (P02VJ) [8]. As shown in Fig. 4A, entry of patient-derived HCVpp P02VJ into Huh7.5.1 cells was efficiently inhibited by anti-CD81 mAb QV-6A8-F2-C4 in a dose-dependent manner. Since we have previously demonstrated that combining anti-receptor mAbs, such as anti-CLDN1 or anti-SR-BI mAbs, with anti-E2 mAb or purified heterologous anti-HCV IgG resulted in a marked synergistic effect [9], [38], we next investigated whether the combination of envelope-specific antibodies and anti-CD81 mAb QV-6A8-F2-C4 also results in an additive or synergistic effect on the inhibition of HCV infection. Thereto, we pre-incubated patient-derived HCVpp with anti-E2 mAb IGH461 or purified heterologous anti-HCV IgG (1 or 10 µg/ml) and studied their ability to inhibit HCVpp entry in cells pre-incubated with increasing concentrations of anti-CD81 mAb QV-6A8-F2-C4. Each antibody was tested alone and in combination to determine the combination index (CI) [38], [43] allowing to conclude about additivity or synergy. As shown in Fig. 4 and Table 1, combination of anti-CD81 and anti-HCV envelope antibodies resulted in a synergistic effect on inhibition of HCVpp P02VJ entry as well as of HCVcc infection decreasing the IC_50_ of anti-CD81 mAb by up to 100-fold. Taken together, these data indicate that targeting CD81 may hold promise for design of novel antiviral strategies targeting both viral and host entry factors.

**Table 1:** Synergistic effect of anti-envelope and anti-CD81 antibodies on inhibition of HCV infection.

Virus	Compound 1	IC_50_ (µg/ml)	Compound 2	IC_50_ for combination (µg/ml)	CI
HCVpp	anti-CD81	2.5±0.3	1 µg/ml anti-E2	1.5±0.06	0.61±0.04
		2.5±0.3	10 µg/ml anti-E2	0.03±0.01	0.16±0.008
		2.5±0.3	1 µg/ml anti-HCV IgG	0.2±0.06	0.75±0.04
		5±0.6*	10 µg/ml anti-HCV IgG	1±0.06*	0.45±0.02
HCVcc	anti-CD81	0.25±0.03	1 µg/ml anti-HCV IgG	0.15±0.03	0.61±0.2
		0.25±0.03	10 µg/ml anti-HCV IgG	0.05±0.006	0.29±0.04

HCVpp of strains P02VJ or HCVcc-Luc Jc1 were pre-incubated with anti-E2 mAb IGH461 or purified heterologous anti-HCV IgG (1 or 10 µg/ml) obtained from an unrelated chronically infected subject or isotype control IgG for 1 hour at 37°C and added to Huh7.5.1 cells pre-incubated with serial dilutions of anti-CD81 QV-6A8-F2-C4 or rat isotype control mAbs. HCVpp entry and HCVcc infection were analyzed by luciferase assay. The Combination Index (CI) was calculated as described [42], [43]. A CI less than 0.9, between 0.9 and 1.1, and more than 1.1 indicates synergy, additivity, and antagonism, respectively. CI for anti-CD81 mAb in combination with 10 µg/ml anti-HCV IgG in HCVpp entry inhibition was calculated for an IC_75_ as the combination resulted in an inhibition below the IC_50_ and is indicated by a star (*). IC_50_ of anti-envelope antibodies: anti-E2, 70±5 µg/ml (for HCVpp); anti-HCV IgG, 40±3 µg/ml (for HCVpp), 120±6 µg/ml (for HCVcc).

### Anti-CD81 monoclonal antibody QV-6A8-F2-C4 inhibits neutralizing antibody-resistant HCV cell-cell transmission and viral dissemination

**Figure 5:**
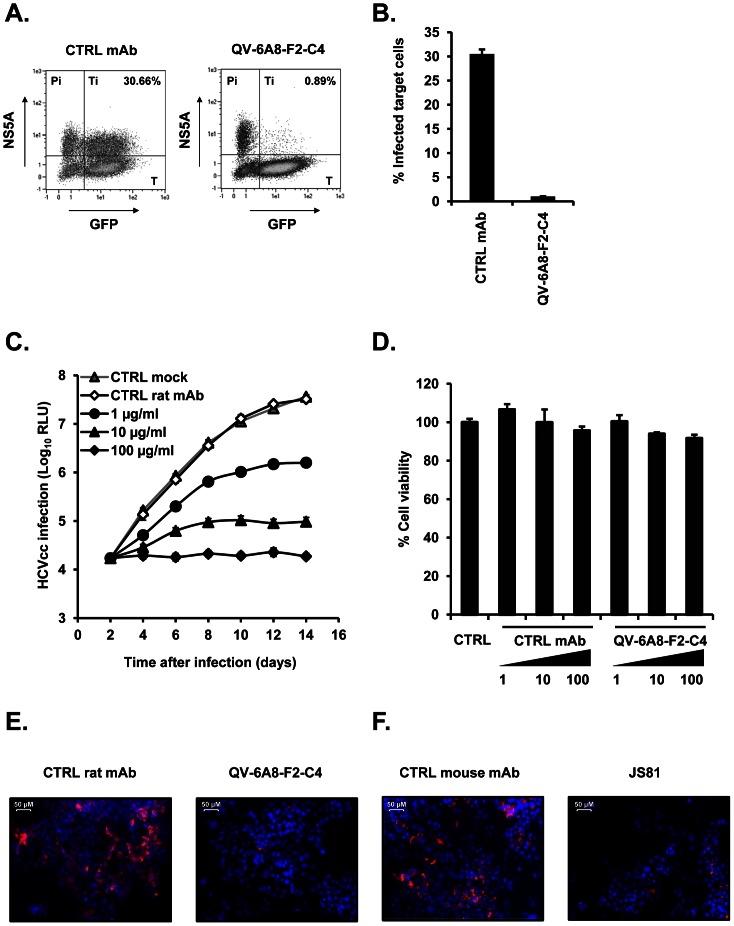
Anti-CD81 mAb inhibits HCV cell-to-cell transmission and viral spread. (A) Quantification of HCV-infected target cells (Ti) after co–cultivation with HCV producer cells (Pi) during incubation with control or anti-CD81 QV-6A8-F2-C4 mAbs (10 µg/ml) in the presence of neutralizing anti-HCV E2 mAb (AP33, 25 µg/ml) by flow cytometry. (B) Percentage of infected target cells is shown as histograms and is represented as means ± SD from three experiments. (C) Long-term analysis of HCVcc infection in the presence or absence of control or anti-CD81 QV-6A8-F2-C4 mAbs at the indicated concentrations. Antibodies were added 48 h after HCVcc infection and control medium or medium containing mAbs were replenished every 4 days. Luciferase activity was determined in cell lysates every 2 days. Data are expressed as Log_10_ RLU and represent means ± SD of three experiments performed in duplicate. (D) Cell viability after long-term exposure to anti-CD81 mAb QV-6A8-F2-C4. Cell viability was assessed using MTT assay after incubation of Huh7.5.1 cells for 14 days in the presence or absence of control or anti-CD81 mAbs at 1, 10, or 100 µg/ml. Data are expressed as % cell viability relative to cells incubated in the absence of mAb and represent means ± SD from one experiment performed in triplicate. (E–F) Virus spread in the presence or absence of anti-CD81 mAbs QV-6A8-F2-C4 (E) and JS81 (F). Antibodies (50 µg/ml) were added 48 h after HCVcc (Jc1) infection and control medium or medium containing antibodies were replenished every 4 days. HCV-infected cells were visualized 9 days post-infection via immunofluorescence using anti-NS5A (E) or anti-E2 (CBH23) (F) antibodies. The percentage of infected cells was calculated as the number of infected cells relative to the total number of cells as assessed by 4′,6-diamidino-2-phenylindole (DAPI) staining of the nuclei.

While cell-free HCV infection is crucial for initiation of infection, direct cell-cell transmission, that is largely resistant to the majority of described neutralizing antibodies, is believed to be most relevant for viral spread and maintenance of infection [10], [32]. To investigate the ability of anti-CD81 mAb QV-6A8-F2-C4 to interfere with neutralizing antibody-resistant viral spread, we used a well-described assay, where cell-free HCV entry is efficiently reduced by more than 90% using a neutralizing anti-E2 mAb, to assess HCV cell-cell transmission [25], [34]. Anti-CD81 mAb QV-6A8-F2-C4 efficiently blocked HCV cell-cell transmission (Fig. 5A–B) indicating that this antibody may prevent viral dissemination *in vitro*. Furthermore, we next assessed whether this anti-CD81 mAb can prevent viral spread when added post-infection. Thereto, cell cultures were first infected with HCV and antibodies were subsequently added to the cells 48 h after infection. Medium or medium supplemented with control mAb or anti-CD81 mAb QV-6A8-F2-C4 was replenished every 4 days until the end of the experiment and HCVcc infection was monitored over 14 days. The anti-CD81 mAb efficiently inhibited HCV spread over 2 weeks in a dose-dependent manner (Fig. 5C) without affecting cell viability as assessed using a MTT test (Fig. 5D). We also assessed Jc1 spread in Huh7.5.1 cells via immunostaining of infected cells after several days of incubation in the presence of anti-CD81 mAbs QV-6A8-F2-C4 and JS81. While 67.6±11.8% of cells incubated with control rat mAb stained positive for NS5A, incubation with QV-6A8-F2-C4 markedly reduced the number of NS5A-positive (7.63±4.8%) cells without displaying any significant cell mortality (Fig. 5E). Incubation of cells with JS81 reduced the number of E2-positive cells (2.3±2.5%) compared with cells incubated with control mouse mAb (60.4±12.5%) (Fig. 5F). However, the number of total cells was significantly and reproducibly reduced in JS81-treated cells which could be due to a cytotoxic or to an anti-proliferative effect. Taken together, these data indicate that anti-CD81 mAb QV-6A8-F2-C4 blocks viral spread by interfering with HCV cell-cell transmission and dissemination without any detectable toxic effect in cell culture models for HCV infection.

## Discussion

In this study we report the successful production of anti-CD81 mAbs using DNA immunization which potently inhibit cell-free HCV infection from different genotypes in a dose-dependent manner and block cell-cell transmission and dissemination. Production of mAbs using DNA immunization has been reported to induce higher avidity antibodies than protein immunization [47], which may be advantageous for the development of antibodies efficiently inhibiting HCV infection. Indeed, among the four anti-CD81 mAbs generated in this study, anti-CD81 mAb QV-6A8-F2-C4 showed very effective inhibition of HCVcc infection and HCVpp entry during HCV-CD81 post-binding interaction(s).

The CD81 LEL has been shown to play an important role in the entry process as soluble recombinant forms of CD81 LEL are able to inhibit HCV infection [12], [13], [15]. The amino acid residues within the CD81 LEL and HCV E2 involved in E2-CD81 binding have been extensively characterized [11], [44], [48], [49], [50]. Interestingly, our studies identify anti-CD81 mAbs with different inhibition profiles on HCV infection. Anti-CD81 mAb QV-6A8-F2-C4 which most efficiently inhibited HCV infection was characterized by binding to cell surface-expressed human CD81 and mutual cross-competition between QV-6A8-F2-C4 and the well-characterized commercially available anti-CD81 antibody JS81 suggests that they recognize similar epitopes on CD81.

These novel anti-CD81 antibodies may be very useful for investigators studying the HCV entry process. Indeed, a panel of antibodies inhibiting HCV entry with different efficacy and recognizing different epitopes is of interest as it may be used to (i) further decipher structural and functional domains in CD81 which are crucial for inhibition and (ii) to more deeply dissect its mechanistic role in the entry process. This will allow a better understanding of CD81 regions binding to envelope glycoprotein E2 or domains involved in the formation of the CD81-CLDN1 complex [22]. Furthermore, the antibodies are useful to study CD81 expression by flow cytometry.

The identification of novel anti-CD81 antibodies may also be relevant for the development of novel antiviral antibodies for prevention and treatment of HCV infection. CD81 may be an attractive therapeutic target for the development of HCV entry inhibitors as it is a key player in the HCV entry process. Small molecules and mAbs targeting CD81 and interfering with HCV infection have previously been described [6]. So far, the effect of the majority of these compounds has been solely assessed on cell-free HCV entry [6]. While cell-free viral entry is undoubtedly essential for initiation of infection, direct cell-cell transmission probably constitutes the dominant mechanism of viral spread and thus persistence of infection [10], [32]. Direct cell-cell transfer has an important impact for the development of antivirals as this process allows viral spreading by escaping extracellular neutralizing antibodies as well as defined antibodies interfering with host cell entry factors [10], [32]. Most of the known HCV entry factors are involved in this process [10], [51]. In addition to CD81-dependent HCV cell-cell transmission, a fraction of viral spread appears to be independent of CD81 [10], [34], [35]. Noteworthy, the anti-CD81 mAb QV-6A8-F2-C4 described in our study not only inhibited cell-free HCV entry but also efficiently and dose-dependently blocked cell-cell transmission and viral spread, providing novel options for the development of efficient anti-HCV therapeutics interfering with this process.

Entry inhibitors, such as anti-CD81 mAbs, are ideal to be applied for the prevention of HCV re-infection in the transplantation setting where currently no clinical option exists to protect HCV-negative transplanted livers from re-infection [3], [4]. An anti-CD81 antibody inhibiting HCV infection *in vitro* has already been demonstrated to prevent HCV infection in the human liver-chimeric Alb-uPA/SCID mouse model [29]. This suggests that targeting CD81 may be an efficient strategy to prevent HCV infection e. g. in transplant recipients where entry has been shown to be a key determinant for infection of the liver graft [6], [8], [46]. In this study, we demonstrate that anti-CD81 mAbs efficiently inhibited the entry of highly infectious HCV escape variants that are resistant to autologous host responses and re-infect the liver graft. Interestingly, combination of HCV envelope-specific antibodies with a CD81-specific mAb resulted in a synergistic activity on the inhibition of HCVcc infection and HCVpp escape variant entry. The combination decreased the concentration needed to achieve a 50% antiviral activity of the individual compounds up to 100-fold. The ability of anti-CD81 mAbs to block entry of HCV escape variants and the marked synergy with anti-envelope antibodies on inhibiting HCV entry indicate that the novel CD81-specific mAbs are prime candidates for prevention of liver graft infection. Furthermore, entry inhibitors may also be efficient antivirals for treatment of HCV infection [52], [53]. Indeed, the ability of anti-CD81 mAb QV-6A8-F2-C4 to block cell-cell transmission and dissemination post-infection without any detectable toxicity suggests that targeting CD81 may also hold promise for the treatment of chronic infection in combination with other antivirals. A potential challenge for the clinical development of anti-CD81 antibodies could be adverse effects. Indeed, CD81 is ubiquitously expressed on the surface of various cell types. Antibodies binding to CD81 may alter the function, expression or signaling of the receptor resulting in side effects. Interestingly, using anti-CD81 mAb QV-6A8-F2-C4, no toxic effects were detected in MTT-based cellular assays (Fig. 5D). However, further *in vivo* studies are needed to address toxicity in hepatic and extrahepatic tissues.

In conclusion, we identified and functionally characterized a novel panel of anti-CD81 mAbs generated by DNA immunization which efficiently inhibit HCV infection and dissemination. These antibodies will be useful for the molecular investigations of virus-host interactions during the HCV entry process and the characterization of CD81 expression in cell lines, primary cells and tissues. Furthermore, one antibody is an interesting and relevant candidate for the development of novel preventive and improved therapeutic antiviral strategies against HCV infection.
